# Self-Assembled AgNP-Containing Nanocomposites Constructed by Electrospinning as Efficient Dye Photocatalyst Materials for Wastewater Treatment

**DOI:** 10.3390/nano8010035

**Published:** 2018-01-10

**Authors:** Yamei Liu, Caili Hou, Tifeng Jiao, Jingwen Song, Xu Zhang, Ruirui Xing, Jingxin Zhou, Lexin Zhang, Qiuming Peng

**Affiliations:** 1 State Key Laboratory of Metastable Materials Science and Technology, Yanshan University, Qinhuangdao 066004, China; liuym@ipe.ac.cn (Y.L.); pengqiuming@ysu.edu.cn (Q.P.); 2 Hebei Key Laboratory of Applied Chemistry, School of Environmental and Chemical Engineering, Yanshan University, Qinhuangdao 066004, China; houcaili@ysu.edu.cn (C.H.); jwsong@ipe.ac.cn (J.S.); zhangxu1991@ysu.edu.cn (X.Z.); rrxing@ipe.ac.cn (R.X.); zhanglexin@ysu.edu.cn (L.Z.); 3 State Key Laboratory of Biochemical Engineering, Institute of Process Engineering, Chinese Academy of Sciences, Beijing 100190, China

**Keywords:** composite membranes, electrospinning, graphene oxide, photocatalyst, dyes degradation, wastewater treatment

## Abstract

The design and self-assembly of graphene oxide (GO)-based composite membranes have attracted enormous attention due to their wide application in nanomaterial and environmental fields. In this work, we have successfully developed a strategy to fabricate new composite membranes based on poly(vinyl alcohol)/poly(acrylic acid)/carboxyl-functionalized graphene oxide modified with silver nanoparticles (PVA/PAA/GO-COOH@AgNPs), which were prepared via thermal treatment and the electrospinning technique. Due to the strong π-π forces and strong electrostatic interactions of GO–COOH sheets, the prepared composite membranes and their lager surface areas were modified by scores of AgNPs, which demonstrated that a high-efficiency photocatalyst removed the organic dyes from the aqueous solutions. The prepared PVA/PAA/GO-COOH@AgNPs nanocomposite membranes showed a remarkable photocatalytic capacity in the catalytic degradation of the methylene blue dye solutions. Most importantly, the whole process was easy, mild, and eco-friendly. Additionally, the as-prepared membranes could be repeatedly used after the catalytic reaction.

## 1. Introduction

In the past few years, with the rapid development of the economy, most factories have been eager to maximize profits at the expense of the environment. In recent years, environmental pollution has drawn public attention because pollution has generated fear for human health and future generations’ ability to live on this planet [1,2]. This is especially true for water pollution, which is mostly attributable to organic dyes released by chemical plants [3,4]. Photocatalysts demonstrate pronounced potential as drivers of chemical reactions when illuminated by sunlight at room temperature [5,6]. Photocatalytic degradation of organic pollutants in water is used as a new wastewater treatment technology, and has become a research hotspot in the wastewater treatment field [7,8]. The current research of photocatalytic technology is mainly concentrated on conventional semiconductor photocatalysts, especially TiO_2_ based materials [7]. Although TiO_2_ has many advantages, such as a wide range of sources and a low price, it can only use the ultraviolet part of the sun. Ultraviolet light accounts for only 5% of solar energy, which limits its practicality. Therefore, finding the catalyst that can respond to visible light, more than 43% of the solar energy, has become the main direction of research [9,10]. It is well known that silver nanoparticles (AgNPs) not only exhibit considerable ultraviolet (UV) light absorption due to the inter-band transition, but also can perform under visible light attributed to the so-called surface plasmon resonance (SPR) effect [11,12]. Hence, silver NPs are promising photocatalysts that exert the whole solar spectrum. Interestingly, it should be noted that various porous TiO_2_-Ag nanostructures, including hollow microspheres and nanotube arrays, have demonstrated enhanced visible light photocatalytic activity for reducing the variety of dyes in water and enhancing water splitting [13,14,15,16,17,18,19,20,21,22,23,24,25,26,27,28].

On the other hand, a nanofibrous composite membrane has the advantage of easy recycling in the field of wastewater adsorption treatment. Electrospinning is considered a simple and versatile technique to fabricate polymeric micro/nanofibers continuously [29,30]. Prepared fibrous materials illustrate many outstanding properties, such as large specific surface area [31,32], favorable porosity [33], and superior controllable thickness [34,35]. These characteristics give the electrospun fibers potential applications as nanocatalysts. Graphene oxide (GO)-based nanocomposite fibers have attracted enormous interest due to their regulated dispersibility, large functional oxygen-containing groups, and preferred reactive positions/sites for special chemical modification [36].

By employing the merit of graphene oxide (GO)-based nanocomposites and many novel advantages of electrospun fibers in present research work, self-assembled electrospun matrical membranes of PVA/PAA/GO–COOH@AgNPs can be processed. The obtained PVA/PAA/GO-COOH membranes were synthesized using thermal treatment and the electrospinning technique while the Ag nanoparticles from the reduction of AgNO_3_ by ascorbic acid solution were firmly immobilized on the surface of the nanofibers with the help of hydrogen bonds and electrostatic interactions. The initial considerations for this experiment follow. First, the prepared PVA/PAA/GO-COOH system was selected as matrix materials for electrospinning due to the chemical and mechanical properties as well as the large specific surface area [37]. Second, compared with the conventional organic solvents, deionized water was used as a solvent to prepare the electrospinning solution, demonstrating low cost and environmentally-friendly features. Finally and most importantly, the Ag nanoparticles showed that they can achieve good stability between the flexible surface of fiber and efficient GO nanosheets, and perform under visible light. The strong π-π forces in GO sheets can attach the powerful adsorption for diverse dyes in water. In addition, the fixed carboxyl group within the GO nanosheet can create strong electrostatic interactions by its highly negative-charged property, which can promote the target dyes’ diffusion and enrichment.

Additionally, the prepared composite membranes acted as high-efficiency catalysts to degrade organic dyes and were easily separated from dye solutions to reuse due to their mechanical strength. This is crucial to the performance evaluation of catalysts. Moreover, presently prepared nanocomposites also have the characteristics of low cost, easy preparation, and environmental friendliness, which demonstrates important potential applications in the catalysis fields.

## 2. Materials and Methods

### 2.1. Materials

Polyvinyl alcohol (PVA, 98–99% hydrolyzed, molecular weight 57,000–66,000) poly(acrylic acid) (PAA, molecular weight ~2000), and chloroacetic acid were provided by Aladdin Reagent (Shanghai, China) and Alfa Aesar Chemicals (Beijing, China). Graphene oxide was synthesized from graphite powder (8000 mesh, 99.95%, Aladdin Reagent) by a modified Hummers method [38]. Rhodamine B (RhB), and methylene blue (MB) were purchased from Tianjin KaiTong Chemical Reagent (Tianjin, China) and used without further purification. Silver nitrate and vitamin C were obtained from Aladdin Reagent (Shanghai, China). Sulfuric acid (H_2_SO_4_, 98%), potassium permanganate (KMnO_4_), potassium nitrate (KNO_3_), hydrogen peroxide (H_2_O_2_, 30%, *w*/*w*), and hydrochloric acid (HCl, 37%) were of analytical reagent grade and were used as received. Deionized (DI) water was used for preparing aqueous solutions in the whole experiment process.

### 2.2. Preparation of Electrospun Composites

**Figure 1:**
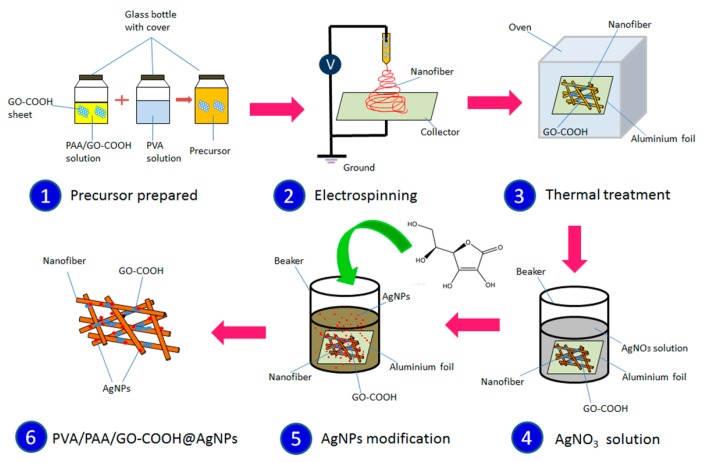
Schematic illustration of the fabrication of PVA/PAA/GO-COOH@AgNPs nanocomposites by electrospinning and thermal treatment.

Graphene oxide (GO), due to its unique two-dimensional single or several-layer sp^2^-bonded carbon sheets, has attracted plenty of research. Other reasons for the interest in graphene oxide include its superior dispersibility and easy functionalization in wide applications. Hence, GO was selected as the key moiety of the self-assembling matrix materials for electrospinning. Silver nanoparticles are promising photocatalysts and are chosen as photocatalysts in present nanocomposites responsible for photocatalytic degradation of organic dyes. The catalytic membranes were prepared via electrospinning of matrix materials and adherence of Ag NPs. As shown in Figure 1, the preparation processes of these composite catalytic membranes were divided into two steps. First, the matrical membranes of PVA/PAA/GO-COOH were prepared through the electrospinning technique and the prepared matrical membranes were insoluble after thermal treatment, which is attributed to the crosslinking reaction between GO-COOH sheets and polymeric nanofibers. Next, the prepared insoluble membranes were modified by Ag nanoparticles when they were immersed into the AgNO_3_ solution with ascorbic acid. The preparation process is very environmentally-friendly, controllable, and easy to operate, which demonstrates potential large-scale applications in dye removal of wastewater.

Carboxyl-functionalized GO (GO-COOH) was first prepared according to a reported method [39], and freeze-dried in low temperature (−50 °C). The 10 wt. % aqueous solution of PVA was prepared by dissolving PVA in deionized water at 80 °C for 12 h under magnetic stirring. PAA solution (30 wt. %) was prepared in deionized water solvent under magnetic stirring at room temperature for 1 h. GO-COOH solid was then added to the above PAA solution and stirred at room temperature for an additional 1 h to obtain the solutions. The above-prepared PVA and PAA/GO-COOH solutions were then mixed with a 1:1 volume ratio and further stirred at room temperature for 1 h to achieve a homogeneous solution for electrospinning [40]. For the electrospinning process, a syringe connected to a stainless steel needle with an inner diameter of 0.6 mm was used to load 10 mL of the above PVA/PAA/GO-COOH precursor solution. During electrospinning, a voltage of 20 kV and a flow rate of 0.5 mL h^−1^ were applied to the spinneret. The produced electrospun nanofiber composite was deposited on a flat aluminum collector placed 15 cm away from the needle, and then dried in a vacuum oven at room temperature for 24 h, as demonstrated in Figure 1. After that, the obtained membrane samples were heated at 120 °C for 3 h to develop a heat-induced crosslinking reaction between carboxyl acid groups in PAA/GO-COOH components and hydroxyl groups in PVA molecules.

The designed PVA/PAA/GO-COOH@AgNPs nanocomposites were prepared by modifying literature reports [41]. After the heating treatment at 120 °C and the crosslinking reaction, PVA/PAA/GO-COOH membranes change to insoluble material in the water through the esterification reaction from carboxylic acid groups and hydroxyl groups. Next, the obtained insoluble composite membranes were dipped into silver nitrate solution with concentration of 25 mg mL^−1^ with mild stirring at room temperature. 1.0 mL of vitamin C solution (15 mg mL^−1^) was added to the above solution. Afterwards, the reduced AgNPs were gradually anchored on the prepared insoluble composite membranes. Thus, the as-prepared PVA/PAA/GO-COOH@AgNPs nanocomposite membranes were regulated by changing the immersed time (30 min, 1 h, 5 h, respectively). After the immersion step, the composite membranes were thoroughly washed with deionized water several times to remove the free/non-adhered AgNPs, and dried in vacuum at 80 °C for 24 h.

### 2.3. Catalytic Tests

To estimate the photocatalytic activity of the PVA/PAA/GO-COOH@AgNPs nanocomposites at room temperature, 30 mg of a freshly prepared PVA/PAA/GO-COOH@AgNPs nanocomposite catalyst was added to 100 mL dye solutions that contains RhB (4 mg mL^−1^) and MB solution (10 mg mL^−1^), respectively. After dispersing catalyst materials using magnetic stirring, the mixed solution was stirred under dark conditions for 30 min to reach adsorption equilibrium state. After that, A UV lamp (7 mW/cm^2^, wavelength range of 360–370 nm; LUYOR-3405; Shanghai LUYOR Co. Ltd., Shanghai, China) was used to irradiate the obtained mixed solution at a 40 cm distance from a light source to liquid surface. The use of a cold light source in a UV lamp can keep the temperature of the solution below 40 °C even after working for 24 h. The dye concentrations in solutions were measured and calculated at different time intervals. For the control group of PVA/PAA/GO-COOH nanocomposite, similar experimental procedures were adopted except for the irradiation of the LV lamp. Upon removal of the solid catalytic samples using the centrifugation process, the supernatant solutions were measured via a 752 UV-vis spectrometer obtained from Sunny Hengping scientific instruments at wavelengths of 632 nm (MB) and 554 nm (RhB) with the pre-established calibration curves, respectively. Thus, the percentage of dye degradation (*K*) was calculated using Equation (1)

(1) $$K=\frac{\left(A_{0}-A_{T}\right)}{A_{T}}*100\%$$

where *K* represents the percentage of degradation; *A*_0_ represents the absorbance of the original solution; *A*_T_ indicates the solution absorbance at any time. In the next recycling experiments, the as-prepared PVA/PAA/GO-COOH@AgNPs nanocomposite membranes (30 mg) were added into 100 mL MB solution (10 mL mL^−1^) under mild stirring. After 4 h of photocatalytic reaction under light irradiation, the composite membrane materials were washed completely with ethanol/deionized water several times. The photocatalytic processes were investigated and repeated for eight consecutive cycles by the same membrane and initial fresh MB solution.

### 2.4. Characterization

A transmission electron microscope instrument (TEM, HT7700, High-Technologies Corporation, Ibaraki, Japan) and a field-emission scanning electron microscopy (FE-SEM, SUPRA 55 SAPPHIRE, Carl Zeiss AG, Oberkochen, Germany) are used for evaluating morphology and observing size, respectively. A SMART LAB X-ray diffractometer (Rigaku, Japan) with a Cu Kα X-ray radiation source performed X-ray diffraction (XRD) characterization. A Raman spectroscopy study was obtained via a confocal Raman microscope (Xplora PLUS, Horiba Jobin Yvon, Edison, NJ, USA) with 532 nm excitation laser and laser power below 1 mW. X-ray photoelectron spectroscopy (XPS) was measured using an ESCALAB 250Xi XPS (Thermo Fisher Scientific, San Jose, CA, USA) with 200 W monochromated Al Kα radiations. Thermogravimetry-differential scanning calorimetry (TG-DSC) analyses were measured using the NETZSCH STA 409 PC Luxxsi multaneous thermal analyzer from Netzsch Instruments Manufacturing Co., Ltd. (Seligenstadt, Germany) in an air conditioned environment.

## 3. Results and Discussion

### 3.1. Characterization of Nanocomposites

**Figure 2:**
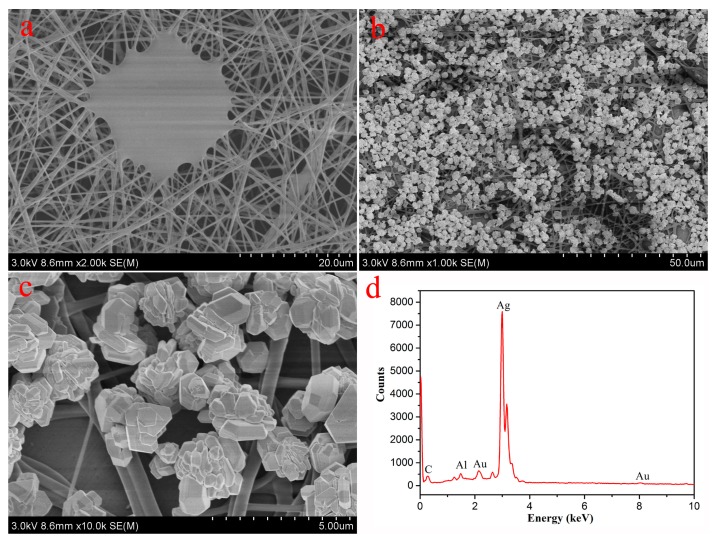
SEM images of the prepared PVA/PAA/GO-COOH nanocomposite by electrospinning and thermal treatment (**a**); and next modified with Ag nanoparticles at 1 h (**b**,**c**); Picture (**d**) indicates the EDXS taken on the Ag nanoparticles shown in (**c**). The Al and Au peaks originate from the aluminum foil substrate and coated Au particles.

**Figure 3:**
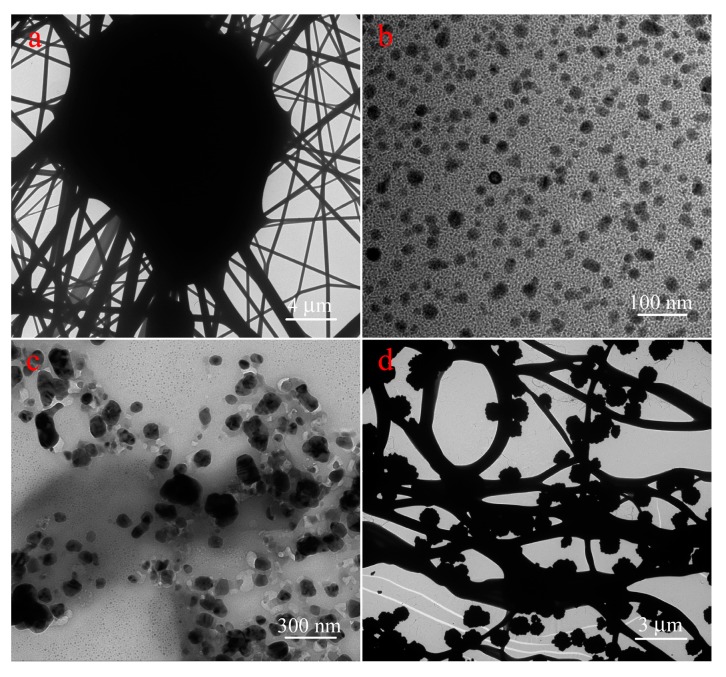
TEM images of the prepared PVA/PAA/GO-COOH nanocomposite (**a**); the obtained Ag nanoparticles at 5 min (**b**) and 1 h (**c**); and PVA/PAA/GO-COOH@AgNPs nanocomposite at a modified time of 1 h (**d**).

First, scanning electron microscopy (SEM) (Figure 2) and transmission electron microscopy (TEM) (Figure 3) characterization were utilized to investigate the morphologies and nanostructures of the prepared pristine PVA/PAA/GO-COOH composite materials. The membrane showed uniform nanostructures with a main fiber diameter distribution of 300–500 nm and a clearly flat structure in the surface of PVA/PAA/GO-COOH nanofibers. This further demonstrated that some GO sheets successfully cross-linked with nanofibers in plane. In addition, the morphologies of nanofiber loaded with silver nanoparticles at incubation time of 1 h are shown in Figure 2b,c. Clearly, the surfaces of PVA/PAA/GO-COOH membranes were modified with lots of Ag nanoparticles stacked onto the fibers’ surface. Energy dispersive X-ray spectroscopy (EDXS) of nanocomposite films (Figure 2d) confirmed the presence of silver on the nanofiber membrane and the specific peak corresponded to the silver metal. Additionally, the size of Ag particles increased in ascorbic acid as time went along until one hour. As shown in Figure 3b,c, the diameters of obtained Ag nanoparticles changed from several tens of nanometers to hundreds of nanometers. Image of Figure 3d indicate that Ag nanoparticles were successfully loaded on the composite membrane with slight aggregation. It should be noted that the synthesized AgNPs in aqueous solution have many hydroxyl groups on the surface of particles. In addition, the environment of the AgNP aqueous solution is neutral, so hydrogen bonds can be expected to form. On the other hand, there are large numbers of carboxyl groups in the PAA molecules. Thus, the obtained nanofiber membranes also have many excess carboxyl groups on the surface. Therefore, AgNPs with many hydroxyl groups can be easily anchored and aggregate together on the surface of prepared nanofibers, mainly due to hydrogen bond interaction.

**Figure 4:**
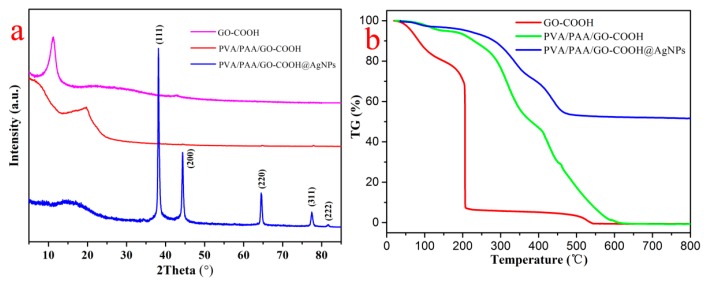
X-ray diffraction patterns (**a**) and TG curves (**b**) of the prepared GO-COOH solid, PVA/PAA/GO-COOH nanocomposite, and PVA/PAA/GO-COOH@AgNPs nanocomposite at modified time of 1 h.

In order to evaluate the strong affiliation between nanocomposite films and silver nanoparticles, X-ray diffraction (XRD) was measured. Figure 4a shows the diffraction patterns of the prepared GO-COOH solid and the PVA/PAA/GO-COOH nanocomposite together with the PVA/PAA/GO-COOH@AgNPs nanocomposite at modified time of 1 h. The 2θ values were observed at 11° (GO-COOH) and 19.7° (PVA/PAA/GO-COOH), suggesting the incorporation of GO component in the obtained composites. At the same time, other characteristic diffraction peaks at 2θ values of 38.2°, 44.3°, 63.5°, and 77.5° conformed with silver corresponding to the (111), (200), (220), and (311) miller indices. This again proves that the nanocomposite membrane is well combined with the silver particles. Thermogravimetry (TG) curves of different materials were also measured to further identify the thermal stability of the membrane under an argon atmosphere, as shown in Figure 4b. The weight losses below 150 °C can be related to the removal of absorbed water while the instant reduction of weight of GO-COOH at 200 °C could be attributed to various oxygen containing chemical groups and alkyl chains within the GO sheets [42,43,44,45,46]. From 200 to 600 °C, the sharp loss of weight could be regarded as the thermal decomposition of the carbon skeleton in the PVA and PAA molecules [47]. With increments of temperature up to 600 °C, the retention quality of PVA/PAA/GO-COOH remained stable. In addition, the weight loss of the PVA/PAA/GO-COOH@AgNPs nanocomposite films was approximately 47.1 wt. % while the GO-COOH and PVA/PAA/GO-COOH nanocomposites lost near 100 wt. %. Therefore, compared to PVA/PAA/GO-COOH, PVA/PAA/GO-COOH@AgNPs composites showed higher thermal stability due to the addition of silver nanoparticles in composite membranes.

**Figure 5:**
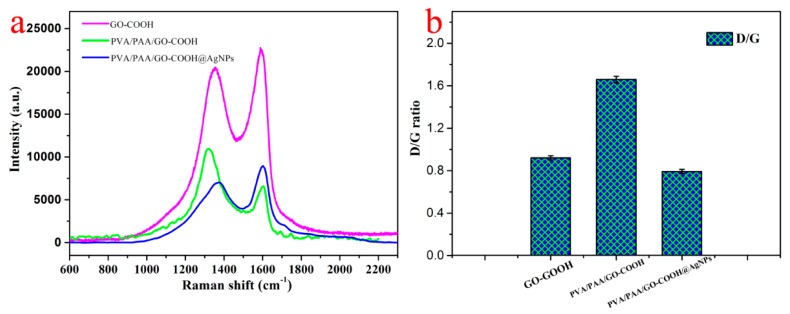
Raman spectroscopy (**a**) and D/G ratios (**b**) of different samples.

In order to investigate the obtained nanofibers containing graphene oxide, we further monitored the prepared composite membrane materials using Raman spectroscopy. It is well known that Raman spectroscopy is the relevant methodology to characterize GO-based composite materials. The measured Raman spectroscopy of prepared nanocomposite materials is shown in Figure 5a. There are two major peaks of graphene sheets in Raman spectra. One band located at 1592 cm^−1^ can be regarded as the G band owing to the in-plane bond-stretching motion of pairs of sp^2^-bonded carbon atoms. Another band at 1354 cm^−1^ mainly originated from the D band, which was lower in intensity than G bands. This shows a broader peak due to the reduction in size of the in-plane sp^2^ domains by the creation of defects, vacancies, and distortions of the sp^2^ domains during oxidation [48]. The Raman spectrum of prepared PVA/PAA/GO-COOH nanofibers displays the characteristic bands of graphene sheets, which confirms that the GO was successfully introduced into the nanofibers during the electrospinning process. In addition, we can also note that the two peaks of PVA/PAA/GO-COOH becomes weaker, which may be attributed to the interaction between GO and PVA/PAA. Furthermore, the D/G peak intensity ratio is used to estimate the sp^2^ domain size in materials containing graphene including sp^2^ and sp^3^ bonds originated from G and D bands [49,50,51,52,53,54,55]. As shown in Figure 5b, the D/G ratio shifted from 0.921 for GO-COOH to 1.66 for PVA/PAA/GO-COOH. This result confirms the successful modification of graphene sheet in the electrospun membrane by esterification reaction between the hydroxyl components in PVA molecules and carboxylic segments in GO-COOH materials as well as the presence of the polymeric alkyl chains linked to GO sheets. The D/G ratio of PVA/PAA/GO-COOH@AgNPs composites decrease to 0.792, which can be due to larger surface area modified by scores of AgNPs.

**Figure 6:**
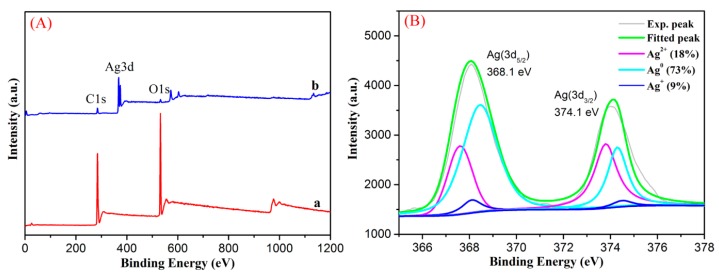
Survey XPS spectra of both samples (**A**): a, PVA/PAA/GO-COOH nanocomposite; b, PVA/PAA/GO-COOH@AgNPs nanocomposite at modified time of 1 h; (**B**) Ag3d deconvolution of XPS peaks in spectra b.

It is important to further investigate interfacial state and composition using X-ray photoelectron spectroscopy (XPS) technique due to the adsorption and photocatalytic application of the as-prepared membrane. Moreover, XPS has been widely used to characterize electronic structures of metal nanoparticles on various substrates [56,57,58,59,60,61,62,63,64,65]. The survey data of XPS spectra from PVA/PAA/GO-COOH and PVA/PAA/GO-COOH@AgNPs nanocomposites was shown in Figure 6A. The peaks of C1s and O1s both appeared in PVA/PAA/GO–COOH and electrospun nanocomposite materials containing the silver while a considerable discrepancy was revealed in the PVA/PAA/GO-COOH@AgNPs membrane using the high resolution silver peak (Ag3d). The membrane should have risen from the silver clusters on the surface. In addition, we analyzed the deconvolution of Ag3d peaks for the PVA/PAA/GO-COOH@AgNPs membranes (Figure 6B). Two strong peaks at ca. 368.1 and 374.1 eV ascribed to Ag(3d_5/2_) and Ag(3d_3/2_) binding energies [66,67,68,69], respectively, were observed. These two bands could be further deconvoluted into three main peaks, which are attributed to the Ag^2+^, Ag^+^, and metallic Ag^0^, respectively. This is consistent with the results of previous reports [67,68]. In addition, according to the data of deconvolution analysis, around 73% of silver were calculated to be in the Ag^0^ state, which confirm the existence of metallic Ag^0^ in the obtained materials and further verifies the successful loading of silver nanoparticles onto the prepared electrospun composite membrane.

### 3.2. Catalytic Performances

**Figure 7:**
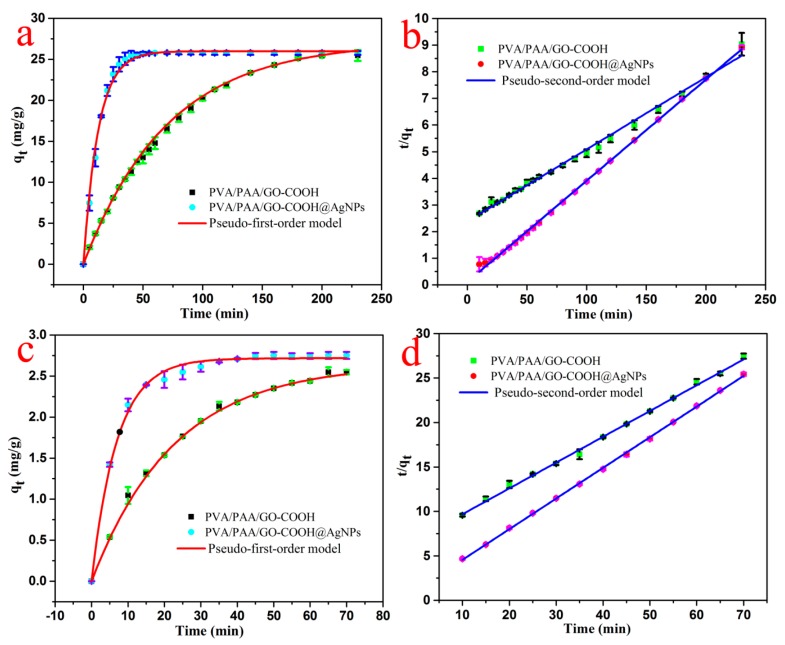
Photocatalytic kinetics curves of as-prepared PVA/PAA/GO-COOH@AgNPs nanocomposite on MB (**a**,**b**) and RhB (**c**,**d**) at 298 K.

In addition, to further assess the dye degradation capacities of the fresh prepared composite membranes, the kinetic experiments of the as-prepared PVA/PAA/GO-COOH@AgNPs nanocomposites were measured and the results are shown in Figure 7. Two different models of dyes, called MB and RhB, were selected to evaluate catalytic performance of the obtained materials. Additionally, the present catalytic experiments were measured and repeated three times. Classical kinetic models were utilized to demonstrate the present catalytic process through the following formulas:

The pseudo-first-order model

(2) $$\log(q_{e}-q_{t})=\log q_{e}-\frac{k}{2.303}t$$

The pseudo-second-order model

(3) $$\frac{t}{q_{t}}=\frac{1}{k\;q_{e}{}^{2}}+\frac{t}{q_{e}}$$

where q_e_ and q_t_ are the dye degraded amount (mg/g) at equilibrium and time t, respectively, and the k_1_ or k_2_ values represent the kinetic rate constants. The kinetic results (Table 1) can be appropriately characterized by either the pseudo-first-order model with a correlation coefficient (R^2^ > 0.9945) or the pseudo-second-order model with a correlation coefficient (R^2^ > 0.9988). Compared to the control group of PVA/PAA/GO-COOH nanocomposite without Ag modification, the removal performances of the model dyes were significantly enhanced in the experiment group.

**Figure 8:**
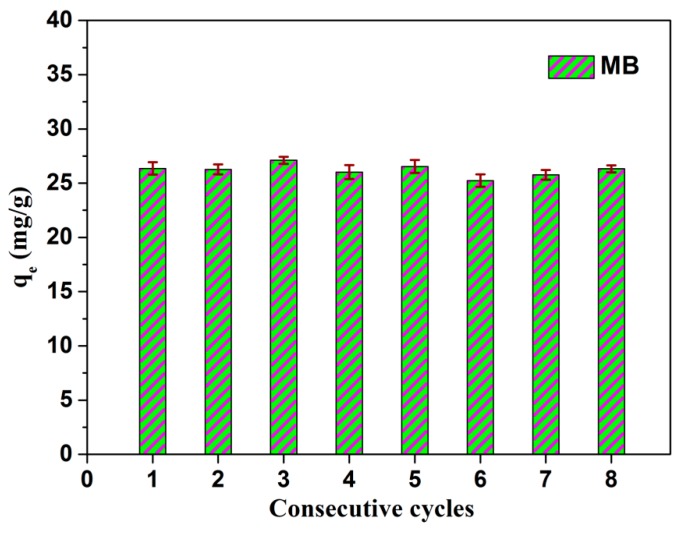
Relative photocatalytic capacity and regeneration studies of as-prepared PVA/PAA/GO-COOH@AgNPs nanocomposite towards MB at room temperature for different consecutive cycles.

On the other hand, good recovery and stability are expected for the large-scale application of composite nanomaterials for wastewater treatment. Here, we investigate the potential application of the obtained PVA/PAA/GO-COOH@AgNPs nanocomposite materials for MB removal. The results indicate that the catalytic amount toward MB stays at about 26.32 mg/g (about 99.8%, compared to 26.36 mg/g in the first catalytic process) for PVA/PAA/GO-COOH@AgNPs nanocomposite membranes after eight consecutive cycles, as presented in Figure 8. In other words, the PVA/PAA/GO-COOH@AgNPs nanocomposite membrane remained vigorous after eight cycles with repeated elution. This demonstrates excellent stability and reutilization of the composite materials [70]. Such outstanding catalytic performance of the GO-COOH@AgNPs membrane benefits from the use of the electrospun membrane as a support for the Ag catalysts. In summary, the prepared composite membrane materials can potentially be applied for wide wastewater treatment.

## 4. Conclusions

Novel multifunctional catalysts of PVA/PAA/GO-COOH@AgNPs nanocomposite membranes have been developed by modifying Ag nanoparticles on electrospun PVA/PAA/GO-COOH nanofibers via an eco-friendly and self-assembled process. The prepared PVA/PAA/GO-COOH nanocomposites provide beneficial support for AgNPs to be loaded on and effectively avoid agglomeration of AgNPs with improved stability for the next catalytic degradation application. Then, the strong π–π forces in GO sheets and the fixed highly negatively-charged carboxyl group within GO nanosheet promote the target dyes’ diffusion and enrichment via the powerful adsorption for diverse dyes and strong electrostatic interactions. For the catalytic degradation of MB, the prepared PVA/PAA/GO-COOH@AgNPs nanocomposite membranes demonstrated significant catalytic activity, even after eight cycles of catalytic degradation at room temperature. The present work thus offers a novel approach for designing and processing new Ag nanoparticle-containing composite materials for catalytic applications in wastewater treatment.

## Figures and Tables

**Table 1:** Kinetic parameters of PVA/PAA/GO-COOH nanocomposite and PVA/PAA/GO-COOH@AgNPs nanocomposite for RhB and MB degradations and removal at 298 K (experimental data from Figure 7).

**RhB**	**Pseudo-First-Order Model**			**Pseudo-Second-Order Model**		
	**q_e_ (mg/g)**	**R^2^**	**K_1_ (min^−1^)**	**q_e_ (mg/g)**	**R^2^**	**K_2_ (g/mg·min)**
PVA/PAA/GO-COOH	2.64	0.9981	0.0442	3.43	0.9976	0.0125
PVA/PAA/GO-COOH@AgNPs	2.71	0.9972	0.1445	2.90	0.9996	0.1072
**MB**	**Pseudo-First-Order Model**			**Pseudo-Second-Order Model**		
	**q_e_ (mg/g)**	**R^2^**	**K_1_ (min^−1^)**	**q_e_ (mg/g)**	**R^2^**	**K_2_ (g/mg·min)**
PVA/PAA/GO-COOH	27.19	0.9989	0.01405	36.97	0.9932	2.07 × 10^−4^
PVA/PAA/GO-COOH@AgNPs	26.01	0.9945	0.07784	26.36	0.9988	1.20 × 10^−2^

## References

[1] Zhang Y.R., Shen S.L., Wang S.Q., Huang J., Su P., Wang Q.R., Zhao B.X. (2014). A dual function magnetic nanomaterial modified with lysine for removal of organic dyes from water solution. Chem. Eng. J..

[2] Ding Q.W., Miao Y.E., Liu T. (2013). Morphology and photocatalytic property of hierarchical polyimide/ZnO fibers prepared via a direct ion-exchange process. ACS Appl. Mater. Interfaces.

[3] Robinson T., McMullan G., Marchant R., Nigam P. (2001). Remediation of dyes in textile effluent: A critical review on current treatment technologies with a proposed alternative. Bioresour. Technol..

[4] Szlinder-Richert J., Usydus Z., Malesa-Ciecwierz M., Polak-Juszczak L., Ruczynska W. (2011). Marine and farmed fish on the Polish market: Comparison of the nutritive value and human exposure to PCDD/Fs and other contaminants. Chemosphere.

[5] Lewis N.S. (2001). Light work with water. Nature.

[6] Zhang M., Chen C., Ma W., Zhao J. (2008). Visible-light-induced Aerobic oxidation of alcohols in a coupled photocatalytic system of dye-sensitized TiO_2_ and TEMPO & dagger. Angew. Chem. Int. Ed..

[7] Kalathil S., Khan M.M., Ansari S.A., Lee J., Cho M.H. (2013). Band gap narrowing of titanium dioxide (TiO_2_) nanocrystals by electrochemically active biofilms and their visible light activity. Nanoscale.

[8] Litter M.I. (1999). Heterogeneous photocatalysis: Transition metal ions in photocatalytic systems. Appl. Catal. B-Environ..

[9] Gratzel M. (2001). Photoelectrochemical cells. Nature.

[10] Asahi R., Morikawa T., Ohwaki T., Aoki K., Taga Y. (2001). Visible-light photocatalysis in nitrogen-doped titanium oxides. Science.

[11] Huang X.H., El-Sayed I.H., Qian W., El-Sayed M.A. (2006). Cancer cell imaging and photothermal therapy in the near-infrared region by using gold nanorods. J. Am. Chem. Soc..

[12] Kiran P.P., Bhaktha B.N.S., Rao D.N., De G. (2004). Nonlinear optical properties and surface-plasmon enhanced optical limiting in Ag–Cu nanoclusters co-doped in SiO_2_ Sol-Gel films. J. Appl. Phys..

[13] Fei J., Li J. (2015). Controlled preparation of porous TiO_2_-Ag nanostructures through supramolecular assembly for plasmon-enhanced photocatalysis. Adv. Mater..

[14] Jiang Z.F., Wei W., Mao D.J., Chen C., Shi Y.F., Lv X.M., Xie J.M. (2015). Silver-loaded nitrogen-doped yolk-shell mesoporous TiO_2_ hollow microspheres with enhanced visible light photocatalytic activity. Nanoscale.

[15] Atarod M., Nasrollahzadeh M., Sajadi S.M. (2016). Euphorbia heterophylla leaf extract mediated green synthesis of Ag/TiO_2_ nanocomposite and investigation of its excellent catalytic activity for reduction of variety of dyes in water. J. Colloid Interface Sci..

[16] Ge M.Z., Cao C.Y., Li S.H., Tang Y.X., Wang L.N., Qi N., Huang J.Y., Zhang K.Q., Al-Deyab S.S., Lai Y.K. (2016). In situ plasmonic Ag nanoparticle anchored TiO_2_ nanotube arrays as visible-light-driven photocatalysts for enhanced water splitting. Nanoscale.

[17] Sung D.I., Kim H.G., Cha S.K., Kim D.H., Lee H.B.R., Kim S.O., Kim D.W., Yeom G.Y. (2017). Photocatalytic effect of Ag/TiO_2_ nanotubes fabricated using 40 nm-scale BCP lithography. Nanosci. Nanotechnol. Lett..

[18] Singh J., Satpati B., Mohapatra S. (2017). Structural, Optical and Plasmonic Properties of Ag-TiO_2_ hybrid plasmonic nanostructures with enhanced photocatalytic activity. Plasmonics.

[19] Wang T., Wei J.X., Shi H.M., Zhou M., Zhang Y., Chen Q., Zhang Z.M. (2017). Preparation of electrospun Ag/TiO_2_ nanotubes with enhanced photocatalytic activity based on water/oil phase separation. Physica E.

[20] Alsharaeh E.H., Bora T., Soliman A., Ahmed F., Bharath G., Ghoniem M.G., Abu-Salah K.M., Dutta J. (2017). Sol-gel-assisted microwave-derived synthesis of anatase Ag/TiO_2_/GO nanohybrids toward efficient visible light phenol degradation. Catalysts.

[21] Prikrylova K., Polievkova E., Drbohlavova J., Vesela M., Hubalek J. (2017). Nanostructured titania decorated with silver nanoparticles for photocatalytic water disinfection. Monatshefte für Chemie-Chemical Monthly.

[22] Zhang L., Wu Z., Li X.L., Wang H.Y., Zhu G. (2017). Novel Ag/TiO_2_ hierarchical hollow spheres composite with enhanced photocatalytic performance. J. Nanosci. Nanotechnol..

[23] Misra M., Singh N., Gupta R.K. (2017). Enhanced visible-light-driven photocatalytic activity of Au@Ag core-shell bimetallic nanoparticles immobilized on electrospun TiO_2_ nanofibers for degradation of organic compounds. Catal. Sci. Technol..

[24] Jia X.H., Dai R.R., Lian D.D., Han S., Wu X.Y., Song H.J. (2017). Facile synthesis and enhanced magnetic, photocatalytic properties of one-dimensional Ag@Fe_3_O_4_-TiO_2_. Appl. Surf. Sci..

[25] Duan Y.Y., Zhang M., Wang L., Wang F., Yang L.P., Li X.Y., Wang C.Y. (2017). Plasmonic Ag-TiO_2−x_ nanocomposites for the photocatalytic removal of NO under visible light with high selectivity: The role of oxygen vacancies. Appl. Catal. B-Environ..

[26] Choi Y., Koo M.S., Bokare A.D., Kim D.H., Bahnemann D.W., Choi W. (2017). Sequential process combination of photocatalytic oxidation and dark reduction for the removal of organic pollutants and Cr(VI) using Ag/TiO_2_. Environ. Sci. Technol..

[27] Zhang L.X., Ni C.H., Jiu H.F., Xie C.M., Yan J.B., Qi G.S. (2017). One-pot synthesis of Ag-TiO_2_/reduced graphene oxide nanocomposite for high performance of adsorption and photocatalysis. Ceram. Int..

[28] Zhang Y., Wang T., Zhou M., Wang Y., Zhang Z.M. (2017). Hydrothermal preparation of Ag-TiO_2_ nanostructures with exposed {001}/{101} facets for enhancing visible light photocatalytic activity. Ceram. Int..

[29] Greiner A., Wendorff J.H. (2007). Electrospinning: A fascinating method for the preparation of ultrathin fibers.. Angew. Chem. Int. Ed..

[30] Bhardwaj N., Kundu S.C. (2010). Electrospinning: A fascinating fiber fabrication technique. Biotechnol. Adv..

[31] Chen M., Patra P.K., Lovett M.L., Kaplan D.L., Bhowmick S. (2009). Role of electrospun fibre diameter and corresponding specific surface area (SSA) on cell attachment. J. Tissue Eng. Regen. Med..

[32] Yan J., Huang Y., Miao Y.E., Weng W.T., Liu T. (2015). Polydopamine-coated electrospun poly(vinyl alcohol)/poly(acrylic acid) membranes as efficient dye adsorbent with good recyclability. J. Hazard. Mater..

[33] Xing R., Wang W., Jiao T., Ma K., Zhang Q., Hong W., Qiu H., Zhou J., Zhang L., Peng Q. (2017). Bioinspired polydopamine sheathed nanofibers containing carboxylate graphene oxide nanosheet for high-efficient dyes scavenger. ACS Sustain. Chem. Eng..

[34] Villarreal-Gómez L.J., Cornejo-Bravo J.M., Vera-Graziano R., Grande D. (2016). Electrospinning as a powerful technique for biomedical applications: A critically selected survey. J. Biomater. Sci. Polym. Ed..

[35] Hou C., Ma K., Jiao T., Xing R., Li K., Zhou J., Zhang L. (2016). Preparation and dye removal capacities of porous silver nanoparticle-containing composite hydrogels via poly(acrylic acid) and silver ions. RSC Adv..

[36] Narayan R., Kim J.E., Kim J.Y., Lee K.E., Kim S.O. (2016). Graphene oxide liquid crystals: Discovery, evolution and applications. Adv. Mater..

[37] Zeng J., Hou H.Q., Wendorff J.H., Greiner A. (2004). Electrospun poly(vinyl alcohol)/poly(acrylic acid) fibres with excellent water-stability. E-Polymers.

[38] Li D., Mueller M.B., Gilje S., Kaner R.B., Wallace G.G. (2008). Processable aqueous dispersions of graphene nanosheets. Nat. Nanotechnol..

[39] Orth E.S., Fonsaca J.E.S., Domingues S.H., Mehl H., Oliveira M.M., Zarbin A.J.G. (2013). Targeted thiolation of graphene oxide and its utilization as precursor for graphene/silver nanoparticles composites. Carbon.

[40] Basturk E., Demir S., Danis O., Kahraman M.V. (2013). Covalent immobilization of a-amylase onto thermally crosslinked electrospun PVA/PAA nanofibrous hybrid membranes. J. Appl. Polym. Sci..

[41] Xu X., Yang Q., Wang Y., Yu H., Chen X., Jing X. (2006). Biodegradable electrospun poly(l-lactide) fibers containing antibacterial silver nanoparticles. Eur. Polym. J..

[42] Konwer S., Boruah R., Dolui S.K. (2011). Studies on conducting polypyrrole/graphene oxide composites as supercapacitor electrode. J. Electron. Mater..

[43] Sharma A., Kumar S., Tripathi B., Singh M., Vijay Y.K. (2009). Aligned CNT/polymer nanocomposite membranes for hydrogen separation. Int. J. Hydrogen Energy.

[44] Xue P., Lu R., Chen G., Zhang Y., Nomoto H., Takafuji M., Ihara H. (2007). Functional organogel based on a salicylideneaniline derivative with enhanced fluorescence emission and photochromism. Chem. Eur. J..

[45] Xing R., Jiao T., Liu Y., Ma K., Zou Q., Ma G., Yan X. (2016). Co-assembly of graphene oxide and albumin/photosensitizer nanohybrids towards enhanced photodynamic therapy. Polymers.

[46] Luo X., Ma K., Jiao T., Xing R., Zhang L., Zhou J., Li B. (2017). Graphene oxide-polymer composite Langmuir films constructed by interfacial thiol-ene photopolymerization. Nanoscale Res. Lett..

[47] Guo R., Jiao T., Xing R., Chen Y., Guo W., Zhou J., Zhang L., Peng Q. (2017). Hierarchical AuNPs-loaded Fe_3_O_4_/polymers nanocomposites constructed by electrospinning with enhanced and magnetically recyclable catalytic capacities. Nanomaterials.

[48] Liu Y., Park M., Shin H.K., Pant B., Choi J., Park Y.W., Lee J.Y., Park S.J., Kim H.-Y. (2014). Facile preparation and characterization of poly(vinyl alcohol)/chitosan/graphene oxide biocomposite nanofibers. J. Ind. Eng. Chem..

[49] Kudin K.N., Ozbas B., Schniepp H.C., Prudhomme R.K., Aksay I.A., Car R. (2008). Raman spectra of graphite oxide and functionalized graphene sheets. Nano Lett..

[50] Kim K.S., Zhao Y., Jang H., Lee S.Y., Kim J.M., Kim K.S., Ahn J.H., Kim P., Choi J.Y., Hong B.H. (2009). Large-scale pattern growth of graphene films for stretchable transparent electrodes. Nature.

[51] Akhavan O. (2015). Bacteriorhodopsin as a superior substitute for hydrazine in chemical reduction of single-layer graphene oxide sheets. Carbon.

[52] Guo R., Jiao T., Li R., Chen Y., Guo W., Zhang L., Zhou J., Zhang Q., Peng Q. (2018). Sandwiched Fe_3_O_4_/carboxylate graphene oxide nanostructures constructed by layer-by-layer assembly for highly efficient and magnetically recyclable dye removal. ACS Sustain. Chem. Eng..

[53] Zhou J., Liu Y., Jiao T., Xing R., Yang Z., Fan J., Liu J., Li B., Peng Q. (2018). Preparation and enhanced structural integrity of electrospun poly(ε-caprolactone)-based fibers by freezing amorphous chains through thiol-ene click reaction. Colloid Surf. A-Physicochem. Eng. Asp..

[54] Zhang R., Xing R., Jiao T., Ma K., Chen C., Ma G., Yan X. (2016). Carrier-free, chemo-photodynamic dual nanodrugs via self-assembly for synergistic antitumor therapy. ACS Appl. Mater. Interfaces.

[55] Huo S., Duan P., Jiao T., Peng Q., Liu M. (2017). Self-assembled luminescent quantum dots to generate full-color and white circularly polarized light. Angew. Chem. Int. Ed..

[56] Zhao X., Ma K., Jiao T., Xing R., Ma X., Hu J., Huang H., Zhang L., Yan X. (2017). Fabrication of hierarchical layer-by-layer assembled diamond based core-shell nanocomposites as highly efficient dye absorbents for wastewater treatment. Sci. Rep..

[57] Guo H., Jiao T., Zhang Q., Guo W., Peng Q., Yan X. (2015). Preparation of graphene oxide-based hydrogels as efficient dye adsorbents for wastewater treatment. Nanoscale Res. Lett..

[58] Li K., Jiao T., Xing R., Zou G., Zhou J., Zhang L., Peng Q. (2018). Fabrication of tunable hierarchical MXene@AuNPs nanocomposites constructed by self-reduction reactions with enhanced catalytic performances. Sci. China Mater..

[59] Jiao T., Ma K., Xing R., Zhang L., Zhou J. (2017). Recent progress on peptide-regulated self-assembly of chromophores nanoarchitectonics and applications. J. YanShan Univ..

[60] Jiao T., Chen K., Zhang L. (2017). Research progress on preparation and application of self-assembled nanofilms. J. YanShan Univ..

[61] Jiao T., Huang X., Zhang L., Zhou J. (2017). Research progress on syntheses of nanomaterials based on photothermal agent/photosensitizer and applications. J. YanShan Univ..

[62] Xing R., Liu K., Jiao T., Zhang N., Ma K., Zhang R., Zou Q., Ma G., Yan X. (2016). An injectable self-assembling collagen-gold hybrid hydrogel for combinatorial antitumor photothermal/photodynamic therapy. Adv. Mater..

[63] Yang W., Yang W., Kong L., Song A., Qin X., Shao G. (2018). Phosphorus-doped 3D hierarchical porous carbon for high-performance supercapacitors: A balanced strategy for pore structure and chemical composition. Carbon.

[64] Yang W., Yang W., Song A., Sun G., Shao G. (2018). 3D interconnected porous carbon nanosheets/carbon nanotubes as a polysulfide reservoir for high performance lithium–sulfur batteries. Nanoscale.

[65] Yang W., Yang W., Song A., Gao L., Sun G., Shao G. (2017). Pyrrole as a promising electrolyte additive to trap polysulfides for lithium-sulfur batteries. J. Power Sources.

[66] Lopez-Salido I., Lim D.C., Kim Y.D. (2005). Ag nanoparticles on highly ordered pyrolytic graphite (HOPG) surfaces studied using STM and XPS. Surf. Sci..

[67] Wang P., Huang B., Qin X., Zhang X., Dai Y., Whangbo M.H. (2009). Ag/AgBr/WO_3_ center dot H_2_O: Visible-light Photocatalyst for Bacteria Destruction. Inorg. Chem..

[68] Wang P., Huang B., Lou Z., Zhang X., Qin X., Dai Y., Zheng Z., Wang X. (2010). Synthesis of highly efficient Ag@AgCl plasmonic photocatalysts with various structures. Chem.-Eur. J..

[69] Wang P., Huang B., Zhang Q., Zhang X., Qin X., Dai Y., Zhan J., Yu J., Liu H., Lou Z. (2010). Highly efficient visible light plasmonic photocatalyst Ag@Ag(Br,I). Chem.-Eur. J..

[70] Song J., Xing R., Jiao T., Peng Q., Yuan C., Möhwald H., Yan X. (2018). Crystalline dipeptide nanobelts based on solid-solid phase transformation self-assembly and their polarization imaging of cells. ACS Appl. Mater. Interfaces.

